# 1-Allyl-6-nitro-1*H*-indazole

**DOI:** 10.1107/S1600536812046478

**Published:** 2012-11-17

**Authors:** Nabil El Brahmi, Mohammed Benchidmi, El Mokhtar Essassi, Sonia Ladeira, Lahcen El Ammari

**Affiliations:** a Laboratoire de Chimie Organique Hétérocyclique URAC21, Faculté des Sciences, Université Mohammed V-Agdal, Avenue Ibn Battouta, BP 1014, Rabat, Morocco; b Laboratoire de Chimie de Coordination, route de Narbonne, 31077 Toulouse, France; c Laboratoire de Chimie du Solide Appliquée, Faculté des Sciences, Université Mohammed V-Agdal, Avenue Ibn Battouta, BP 1014, Rabat, Morocco

## Abstract

The fused five- and six-membered rings in the title mol­ecule, C_10_H_9_N_3_O_2_, are essentially coplanar, the largest deviation from the mean plane being 0.012 (1) Å for the C atom linked to the nitro group. The fused-ring system makes a dihedral angle of 11.34 (6)° with the nitro group, leading to a syn-periplanar conformation. The plane through the atoms forming the allyl group is nearly perpendicular to the indazole fused-ring system, as indicated by the dihedral angle of 73.3 (5)°. In the crystal, each mol­ecule is linked to its symmetry equivalent about the center of inversion by pairs of non-classical C—H⋯O hydrogen bonds, forming an extended tape motif parallel to the (-12-1) plane.

## Related literature

For the pharmacological and biochemical properties of substituted indazoles, see: Saczewski *et al.* (2008 ▶); Jones *et al.* (2009 ▶); Bouissane *et al.* (2006 ▶). For compounds with similar structures, see: El Brahmi *et al.* (2009 ▶, 2011 ▶).
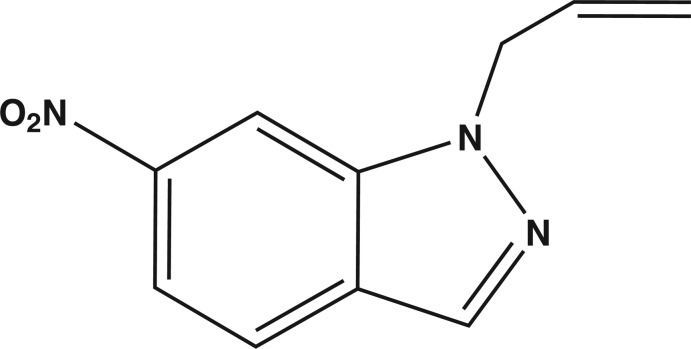


## Experimental

### 

#### Crystal data

C_10_H_9_N_3_O_2_
*M* *_r_* = 203.20
Triclinic, 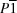

*a* = 4.3630 (16) Å
*b* = 8.3245 (7) Å
*c* = 13.541 (5) Å
α = 95.647 (2)°
β = 98.46 (2)°
γ = 97.770 (2)°
*V* = 478.5 (3) Å^3^
*Z* = 2
Mo *K*α radiation
μ = 0.10 mm^−1^
*T* = 296 K
0.38 × 0.29 × 0.27 mm

#### Data collection

Bruker Kappa APEXII Quazar area-detector diffractometer
Absorption correction: multi-scan (*SADABS*; Bruker, 2009 ▶) *T*_min_ = 0.957, *T*_max_ = 0.997
8258 measured reflections
2109 independent reflections
1675 reflections with *I* > 2σ(*I*)
*R*_int_ = 0.021

#### Refinement

*R*[*F* ^2^ > 2σ(*F* ^2^)] = 0.034
*wR*(*F* ^2^) = 0.100
*S* = 1.06
2109 reflections
136 parameters
H-atom parameters constrained
Δρ_max_ = 0.18 e Å^−3^
Δρ_min_ = −0.20 e Å^−3^

### 

Data collection: *APEX2* (Bruker, 2009 ▶); cell refinement: *SAINT* (Bruker, 2009 ▶); data reduction: *SAINT*; program(s) used to solve structure: *SHELXS97* (Sheldrick, 2008 ▶); program(s) used to refine structure: *SHELXL97* (Sheldrick, 2008 ▶); molecular graphics: *ORTEP-3 for Windows* (Farrugia, 2012 ▶) and *Mercury* (Macrae *et al.* 2008 ▶); software used to prepare material for publication: *PLATON* (Spek, 2009 ▶) and *publCIF* (Westrip, 2010 ▶).

## Supplementary Material

Crystal structure: contains datablock(s) I, global. DOI: 10.1107/S1600536812046478/pk2452sup1.cif

Structure factors: contains datablock(s) I. DOI: 10.1107/S1600536812046478/pk2452Isup2.hkl

Supplementary material file. DOI: 10.1107/S1600536812046478/pk2452Isup3.cml

Additional supplementary materials:  crystallographic information; 3D view; checkCIF report

## Figures and Tables

**Table 1:** Hydrogen-bond geometry (Å, °)

*D*—H⋯*A*	*D*—H	H⋯*A*	*D*⋯*A*	*D*—H⋯*A*
C6—H6⋯O1^i^	0.93	2.51	3.3973 (17)	160
C8—H8*A*⋯O1^i^	0.97	2.53	3.4475 (19)	157
C2—H2⋯O2^ii^	0.93	2.66	3.3911 (17)	136

Symmetry codes: (i) 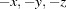; (ii) 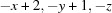.

**Fig. 1.**
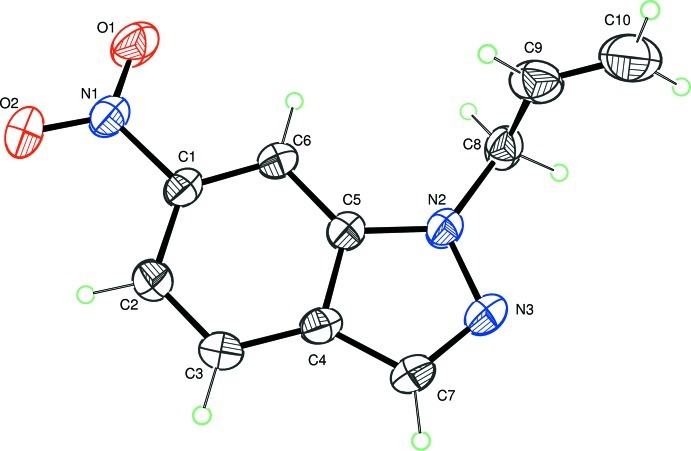
Molecular structure of the title compound, with displacement ellipsoids drawn at the 50% probability level. H atoms are represented as small circles.

**Fig. 2.**
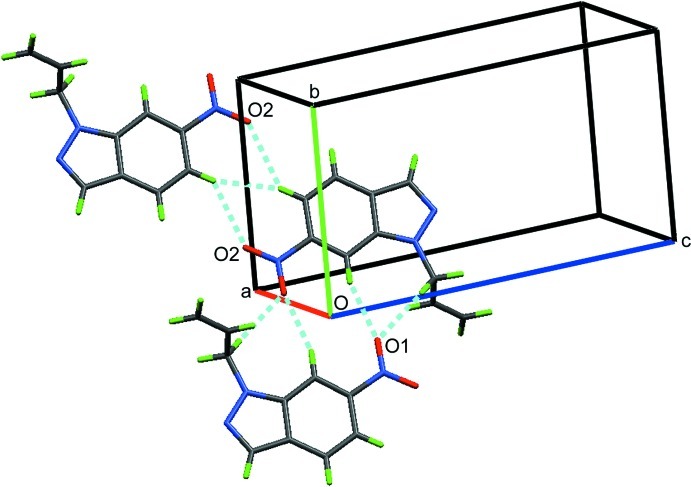
The title molecule and its symmetry equivalent through the inversion center linked by hydrogen bonds and building extended tape motifs parallel to the plane (-1 2 -1).

## supplementary crystallographic information

### Comment

Indazole derivatives constitute an exciting heterocyclic family because of their important biological activities. Thus substituted indazoles are generally found to be of pharmaceutical interest in a variety of therapeutic areas (Saczewski *et al.*, 2008, Jones *et al.*, 2009) and with significant cytotoxicities against human (colon and prostate) and murine (leukemia) cell lines (Bouissane *et al.*, 2006).

The plot of the structure of the title compound shows the indazole ring system is linked to a C_3_H_5_ chain and to a nitro group (Fig.1). The fused-ring system is essentially planar, with a maximum deviation of 0.012 (1) Å for C1. The allyl group is nearly perpendicular to indazole plane as indicated by the torsion angle of C9 C8 N2 N3 = 88.35 (18)°. Moreover, the dihedral angle of 11.34 (6)° between the fused ring system and the nitro group lead to a synperiplanar conformation. The structure of the 1-Allyl-6-nitro-indazole is similar to that reported for the following molecules: 1-allyl-3-chloro-6-nitro-1*H*-indazole and 3-bromo-6-nitro-1-(prop-2-ynyl)-1*H*-indazole (El Brahmi *et al.* 2009, 2011).

In the crystal, each molecule and its symmetry equivalent through the inversion center are linked by C6–H6···O1, C8–H8B···O1 and C2–H2···O2, non-classical hydrogen bonds, which results in an extended tape motif parallel to the plane (-1 2 -1) as shown in Fig.2.

### Experimental

6-nitroindazole (5 mmol) and allyl bromide (10 mmol) were reacted in THF (40 ml) in the presence of potassium carbonate (10 mmol) and tetra-n-butylammonium bromide (0.5 mmol). The mixture was stirred for 24 h, filtered, and the THF removed under vacuum. The product was separated by chromatography on silica gel with a hexane:ethyl acetate (9:1) solvent system. The compound was obtained as yellow crystals in 68% yield.

### Refinement

H atoms were located in a difference map and treated as riding with N—H = 0.86 Å, C—H = 0.93 Å (aromatic), and C—H = 0.97 Å (methylene). with *U*_iso_(H) = 1.2 *U*_eq_ (aromatic, methylene).

### Crystal data

**Table:**

C_10_H_9_N_3_O_2_	*Z* = 2
*M**_r_* = 203.20	*F*(000) = 212
Triclinic, *P*1	*D*_x_ = 1.410 Mg m^−^^3^
Hall symbol: -P 1	Mo *K*α radiation, λ = 0.71073 Å
*a* = 4.3630 (16) Å	Cell parameters from 2109 reflections
*b* = 8.3245 (7) Å	θ = 3.7–27.1°
*c* = 13.541 (5) Å	µ = 0.10 mm^−^^1^
α = 95.647 (2)°	*T* = 296 K
β = 98.46 (2)°	Block, yellow
γ = 97.770 (2)°	0.38 × 0.29 × 0.27 mm
*V* = 478.5 (3) Å^3^	

### Data collection

**Table:**

Bruker Kappa APEXII Quazar area-detector diffractometer	2109 independent reflections
Radiation source: microfocus sealed tube	1675 reflections with *I* > 2σ(*I*)
Multilayer optics monochromator	*R*_int_ = 0.021
φ and ω scans	θ_max_ = 27.1°, θ_min_ = 3.7°
Absorption correction: multi-scan (*SADABS*; Bruker, 2009)	*h* = −5→5
*T*_min_ = 0.957, *T*_max_ = 0.997	*k* = −10→10
8258 measured reflections	*l* = −17→17

### Refinement

**Table:**

Refinement on *F*^2^	Primary atom site location: structure-invariant direct methods
Least-squares matrix: full	Secondary atom site location: difference Fourier map
*R*[*F*^2^ > 2σ(*F*^2^)] = 0.034	Hydrogen site location: difference Fourier map
*wR*(*F*^2^) = 0.100	H-atom parameters constrained
*S* = 1.06	*w* = 1/[σ^2^(*F*_o_^2^) + (0.0593*P*)^2^ + 0.0394*P*] where *P* = (*F*_o_^2^ + 2*F*_c_^2^)/3
2109 reflections	(Δ/σ)_max_ < 0.001
136 parameters	Δρ_max_ = 0.18 e Å^−^^3^
0 restraints	Δρ_min_ = −0.20 e Å^−^^3^

### Special details

**Table:**

Geometry. All e.s.d.'s (except the e.s.d. in the dihedral angle between two l.s. planes) are estimated using the full covariance matrix. The cell e.s.d.'s are taken into account individually in the estimation of e.s.d.'s in distances, angles and torsion angles; correlations between e.s.d.'s in cell parameters are only used when they are defined by crystal symmetry. An approximate (isotropic) treatment of cell e.s.d.'s is used for estimating e.s.d.'s involving l.s. planes.
Refinement. Refinement of *F*^2^ against all reflections. The weighted *R*-factor *wR* and goodness of fit *S* are based on *F*^2^, conventional *R*-factors *R* are based on *F*, with *F* set to zero for negative *F*^2^. The threshold expression of *F*^2^ > 2σ(*F*^2^) is used only for calculating *R*-factors(gt) *etc*. and is not relevant to the choice of reflections for refinement. *R*-factors based on *F*^2^ are statistically about twice as large as those based on *F*, and *R*- factors based on all data will be even larger.

### Fractional atomic coordinates and isotropic or equivalent isotropic displacement parameters (Å^2^)

**Table:**

	*x*	*y*	*z*	*U*_iso_*/*U*_eq_	
C1	0.5250 (3)	0.28071 (13)	0.07294 (8)	0.0285 (3)	
C2	0.7333 (3)	0.42576 (14)	0.10756 (9)	0.0336 (3)	
H2	0.8493	0.4767	0.0639	0.040*	
C3	0.7649 (3)	0.49196 (14)	0.20582 (9)	0.0347 (3)	
H3	0.9001	0.5887	0.2297	0.042*	
C4	0.5890 (3)	0.41073 (13)	0.26971 (9)	0.0296 (3)	
C5	0.3813 (3)	0.26597 (13)	0.23161 (8)	0.0269 (3)	
C6	0.3433 (3)	0.19722 (13)	0.13128 (8)	0.0279 (3)	
H6	0.2053	0.1020	0.1061	0.034*	
C7	0.5573 (3)	0.43449 (15)	0.37259 (9)	0.0357 (3)	
H7	0.6669	0.5214	0.4182	0.043*	
C8	0.0238 (3)	0.06785 (15)	0.31003 (9)	0.0337 (3)	
H8A	−0.1083	0.0416	0.2447	0.040*	
H8B	−0.1104	0.0882	0.3595	0.040*	
C9	0.1822 (3)	−0.07536 (15)	0.33350 (11)	0.0430 (3)	
H9	0.3288	−0.1040	0.2942	0.052*	
C10	0.1274 (4)	−0.16305 (18)	0.40592 (12)	0.0599 (4)	
H10A	0.2369	−0.2555	0.4167	0.072*	
H10B	−0.0296	−0.1344	0.4488	0.072*	
N1	0.5029 (2)	0.21201 (12)	−0.03272 (7)	0.0335 (3)	
N2	0.2434 (2)	0.21521 (11)	0.30948 (7)	0.0306 (2)	
N3	0.3528 (3)	0.31791 (12)	0.39570 (7)	0.0360 (3)	
O1	0.2915 (2)	0.09935 (12)	−0.06728 (7)	0.0468 (3)	
O2	0.6971 (3)	0.26879 (12)	−0.08121 (7)	0.0515 (3)	

### Atomic displacement parameters (Å^2^)

**Table:**

	*U*^11^	*U*^22^	*U*^33^	*U*^12^	*U*^13^	*U*^23^
C1	0.0332 (6)	0.0299 (5)	0.0236 (6)	0.0101 (5)	0.0050 (5)	0.0012 (4)
C2	0.0385 (7)	0.0296 (6)	0.0340 (6)	0.0042 (5)	0.0099 (5)	0.0051 (5)
C3	0.0399 (7)	0.0263 (5)	0.0361 (7)	0.0005 (5)	0.0068 (5)	0.0003 (5)
C4	0.0342 (6)	0.0265 (5)	0.0271 (6)	0.0064 (5)	0.0028 (5)	−0.0015 (4)
C5	0.0279 (5)	0.0280 (5)	0.0255 (6)	0.0073 (4)	0.0036 (4)	0.0031 (4)
C6	0.0290 (6)	0.0276 (5)	0.0257 (6)	0.0046 (4)	0.0018 (4)	−0.0008 (4)
C7	0.0429 (7)	0.0339 (6)	0.0266 (6)	0.0020 (5)	0.0029 (5)	−0.0044 (5)
C8	0.0300 (6)	0.0421 (6)	0.0261 (6)	−0.0028 (5)	0.0041 (5)	0.0023 (5)
C9	0.0370 (7)	0.0356 (7)	0.0521 (8)	−0.0037 (5)	0.0074 (6)	−0.0032 (6)
C10	0.0817 (12)	0.0388 (7)	0.0518 (9)	0.0053 (8)	−0.0079 (8)	0.0024 (7)
N1	0.0409 (6)	0.0351 (5)	0.0263 (5)	0.0117 (4)	0.0070 (4)	0.0021 (4)
N2	0.0343 (5)	0.0336 (5)	0.0223 (5)	0.0018 (4)	0.0048 (4)	0.0005 (4)
N3	0.0426 (6)	0.0387 (6)	0.0240 (5)	0.0034 (5)	0.0036 (4)	−0.0031 (4)
O1	0.0512 (6)	0.0529 (6)	0.0297 (5)	−0.0007 (5)	0.0020 (4)	−0.0088 (4)
O2	0.0659 (7)	0.0549 (6)	0.0364 (5)	0.0031 (5)	0.0255 (5)	0.0017 (4)

### Geometric parameters (Å, º)

**Table:**

C1—C6	1.3706 (16)	C7—H7	0.9300
C1—C2	1.4036 (17)	C8—N2	1.4522 (15)
C1—N1	1.4720 (15)	C8—C9	1.4942 (18)
C2—C3	1.3691 (17)	C8—H8A	0.9700
C2—H2	0.9300	C8—H8B	0.9700
C3—C4	1.4016 (16)	C9—C10	1.309 (2)
C3—H3	0.9300	C9—H9	0.9300
C4—C5	1.4087 (15)	C10—H10A	0.9711
C4—C7	1.4174 (17)	C10—H10B	0.9983
C5—N2	1.3631 (14)	N1—O2	1.2203 (13)
C5—C6	1.3985 (16)	N1—O1	1.2256 (14)
C6—H6	0.9300	N2—N3	1.3605 (14)
C7—N3	1.3204 (16)		
			
C6—C1—C2	124.41 (11)	C4—C7—H7	124.2
C6—C1—N1	117.64 (10)	N2—C8—C9	112.95 (10)
C2—C1—N1	117.95 (10)	N2—C8—H8A	109.0
C3—C2—C1	119.75 (11)	C9—C8—H8A	109.0
C3—C2—H2	120.1	N2—C8—H8B	109.0
C1—C2—H2	120.1	C9—C8—H8B	109.0
C2—C3—C4	118.53 (11)	H8A—C8—H8B	107.8
C2—C3—H3	120.7	C10—C9—C8	124.06 (14)
C4—C3—H3	120.7	C10—C9—H9	118.0
C3—C4—C5	119.81 (11)	C8—C9—H9	118.0
C3—C4—C7	136.30 (11)	C9—C10—H10A	119.9
C5—C4—C7	103.89 (10)	C9—C10—H10B	119.2
N2—C5—C6	130.53 (10)	H10A—C10—H10B	120.9
N2—C5—C4	106.94 (10)	O2—N1—O1	123.28 (10)
C6—C5—C4	122.53 (10)	O2—N1—C1	118.53 (10)
C1—C6—C5	114.96 (10)	O1—N1—C1	118.18 (10)
C1—C6—H6	122.5	N3—N2—C5	111.12 (10)
C5—C6—H6	122.5	N3—N2—C8	120.52 (9)
N3—C7—C4	111.61 (11)	C5—N2—C8	128.23 (9)
N3—C7—H7	124.2	C7—N3—N2	106.44 (10)
			
C6—C1—C2—C3	0.27 (18)	N2—C8—C9—C10	125.37 (14)
N1—C1—C2—C3	−178.80 (10)	C6—C1—N1—O2	−168.29 (10)
C1—C2—C3—C4	0.77 (17)	C2—C1—N1—O2	10.84 (16)
C2—C3—C4—C5	−1.17 (16)	C6—C1—N1—O1	11.05 (16)
C2—C3—C4—C7	178.55 (13)	C2—C1—N1—O1	−169.83 (10)
C3—C4—C5—N2	−179.75 (10)	C6—C5—N2—N3	178.95 (11)
C7—C4—C5—N2	0.45 (12)	C4—C5—N2—N3	−0.67 (12)
C3—C4—C5—C6	0.59 (16)	C6—C5—N2—C8	3.07 (19)
C7—C4—C5—C6	−179.21 (10)	C4—C5—N2—C8	−176.55 (11)
C2—C1—C6—C5	−0.84 (16)	C9—C8—N2—N3	−88.19 (13)
N1—C1—C6—C5	178.23 (9)	C9—C8—N2—C5	87.34 (14)
N2—C5—C6—C1	−179.17 (10)	C4—C7—N3—N2	−0.31 (14)
C4—C5—C6—C1	0.40 (15)	C5—N2—N3—C7	0.62 (13)
C3—C4—C7—N3	−179.84 (13)	C8—N2—N3—C7	176.85 (10)
C5—C4—C7—N3	−0.09 (13)		

### Hydrogen-bond geometry (Å, º)

**Table:**

*D*—H···*A*	*D*—H	H···*A*	*D*···*A*	*D*—H···*A*
C6—H6···O1^i^	0.93	2.51	3.3973 (17)	160
C8—H8*A*···O1^i^	0.97	2.53	3.4475 (19)	157
C2—H2···O2^ii^	0.93	2.66	3.3911 (17)	136

Symmetry codes: (i) −*x*, −*y*, −*z*; (ii) −*x*+2, −*y*+1, −*z*.
